# Clinical Practice and Diagnostic Confidence Regarding Pediatric Oral Mucosal Lesions Among Dentists, Pediatricians, and General Practitioners: A Cross-Sectional Study

**DOI:** 10.3390/pediatric18020033

**Published:** 2026-03-02

**Authors:** Karmela Dzaja, Lidia Gavic, Ana Glavina, Marija Badrov, Danijela Delic Vukic, Livia Sukanec, Antonija Tadin

**Affiliations:** 1 Department of Restorative Dental Medicine and Endodontics, Study of Dental Medicine, School of Medicine, University of Split, 21000 Split, Croatia; karmela.dzaja@mefst.hr (K.D.); lgavic@mefst.hr (L.G.); marija.badrov@mefst.hr (M.B.); 2 Department of Oral Medicine, Study of Dental Medicine, School of Medicine, University of Split, 21000 Split, Croatia; glavina2014@gmail.com (A.G.); livia.sukanec@mefst.hr (L.S.); 3 Department of Dental Medicine, University Hospital of Split, 21000 Split, Croatia; 4 Split-Dalmatia County Health Center, Kavanjinova 2, 21000 Split, Croatia; danijelastdelic@gmail.com

**Keywords:** oral mucosal diseases, pediatric dentistry, primary health care, diagnostic confidence, pediatricians, general practitioners, dentists

## Abstract

Background: Pediatric oral mucosal lesions are common and may indicate local or systemic disease, yet their recognition in primary healthcare often depends on non-dental professionals. Aim: To assess the preparedness of dentists, pediatricians, and family/general practitioners for pediatric oral mucosal conditions based on self-assessed diagnostic confidence, clinical management, and referral behavior. Methods: An online cross-sectional survey was conducted among 632 primary healthcare professionals (dentists: n = 262; family/general practitioners: n = 278; pediatricians: n = 92). The questionnaire assessed clinical exposure, self-assessed knowledge, diagnostic confidence, management practices, and referral patterns. Data were analyzed using chi-square or Fisher’s exact test and the Kruskal–Wallis test (*p* < 0.05). Results: Dentists reported significantly higher self-assessed knowledge and diagnostic confidence than pediatricians and family/general practitioners (*p* < 0.001). Good self-assessed knowledge of pediatric oral health was reported by 26.3% of dentists, compared with 7.9% of family/general practitioners and 6.5% of pediatricians. While most pediatricians (80.4%) and family/general practitioners (77.0%) reported routinely examining the oral cavity in children, independent treatment of oral mucosal lesions was more frequently reported by dentists (75.2%) than by pediatricians (52.2%) or family/general practitioners (70.9%) (*p* < 0.001). Referral patterns differed between groups, and willingness to attend future pediatric oral health education was high across all professionals (75.0–84.2%). Conclusions: Dentists demonstrated higher diagnostic confidence in pediatric oral mucosal lesions than pediatricians and family/general practitioners, who more often relied on referral. These findings support the value of targeted education and strengthened interdisciplinary collaboration in primary pediatric healthcare.

## 1. Introduction

Oral mucosal lesions are a common clinical finding in the pediatric population and include a wide range of changes with diverse etiologies, such as inflammatory, infectious, traumatic, developmental, and reactive lesions. Data from various countries indicate that oral mucosal lesions are frequently encountered in children, with considerable variability in prevalence across age groups and regions [1,2,3]. In Croatia, data on oral mucosal lesions in children are limited and largely derived from dental clinical or tertiary care settings. Available studies report a prevalence of approximately 5.2%, with primary herpetic gingivostomatitis, recurrent aphthous ulceration, geographic tongue, and trauma-related lesions being the most frequently observed conditions [4,5].

Due to the unique characteristics of the developing immune system, pathological conditions of the oral cavity in children often differ in etiology from those seen in adults, making early diagnosis and timely local or systemic treatment especially important. Although many oral mucosal lesions appear clinically benign, they can significantly impair a child’s quality of life by causing pain, discomfort, and difficulties with feeding, speech, and sleep [6,7,8]. Additionally, oral mucosal changes may be early clinical manifestations of systemic diseases, nutritional deficiencies, or immunological disorders. Prompt identification and accurate differential diagnosis are therefore crucial for preventing complications and reducing the need for more invasive therapeutic interventions at later stages [8,9].

Managing oral mucosal lesions in children requires a multidisciplinary approach involving several healthcare professionals [1]. Dentists—especially specialists in pediatric dentistry and oral medicine—play a central role in diagnosis and treatment, but their involvement should not occur in isolation [10,11,12,13,14,15,16,17,18,19]. Pediatricians and family or general practitioners are often the first healthcare providers to see children and their caregivers, placing them in a key position for early recognition of oral mucosal changes and other oral and dental problems [20,21,22,23,24]. However, the oral cavity is often not systematically examined during routine pediatric visits, which can lead to delayed diagnosis, inappropriate management, or unnecessary referrals. Enhancing the knowledge and diagnostic confidence of pediatricians and family physicians is essential to improve clinical outcomes, optimize referral pathways, and ensure efficient use of healthcare resources [24,25,26,27]. Education on oral health in medical curricula for pediatricians and family physicians is often limited and primarily focused on dental diseases, with less emphasis on oral mucosal lesions. This limitation is also evident in Croatia, where oral health education within medical training remains limited and largely non-preventive in scope. At the undergraduate level, all four Croatian medical schools (Zagreb, Rijeka, Split, and Osijek) include a mandatory course in Maxillofacial Surgery and Dental Medicine. This course primarily focuses on surgical pathology and treatment and does not include structured training in preventive dentistry, oral health promotion, or routine clinical oral health care. In pediatric specialty training, dental medicine is addressed only briefly through a short, seven-day Pediatric Dental Medicine course, and oral health is not defined as a longitudinal competency within the pediatric residency curriculum. In contrast, family medicine residency programs in Croatia do not include dental medicine or oral health as part of mandatory courses or core competencies [28,29]. In contrast, dental education programs provide detailed theoretical and practical training in oral mucosal pathology. This educational gap may lead to uncertainty in clinical practice and increased reliance on referrals, even in cases where basic management could be effectively provided in primary healthcare settings [20,30,31,32,33,34]. Given the frequency and clinical relevance of oral mucosal lesions in children, there is a clear need for enhanced education and interdisciplinary collaboration. Strengthening cooperation among dentists, pediatricians, and family or general practitioners, along with integrating pediatric oral health into continuing medical education, could improve early detection, appropriate referral, and management of children with oral mucosal changes, thereby contributing to more comprehensive healthcare delivery and better protection of both oral and general health in the pediatric population [29,32,35].

Although previous studies have examined oral mucosal lesions in children and assessed diagnostic knowledge or confidence primarily among dental professionals and, to a lesser extent, pediatricians, there is a marked lack of evidence focusing on family/general practitioners [4,5,12,18,19]. To date, no studies have specifically evaluated preparedness, diagnostic confidence, clinical exposure, and referral practices related to pediatric oral mucosal lesions among family physicians and pediatricians working in primary healthcare. Therefore, this cross-sectional study aimed to assess the level of preparedness of dentists, pediatricians, and family/general practitioners in recognizing and managing pediatric oral mucosal conditions, with particular emphasis on self-assessed diagnostic confidence, frequency of clinical encounters and treatment, and referral practices when independent management was not undertaken. It was hypothesized that dentists would demonstrate higher diagnostic confidence and greater involvement in the management of pediatric oral mucosal lesions compared with pediatricians and family/general practitioners, while family physicians would report the lowest diagnostic confidence and the highest reliance on referral pathways.

## 2. Materials and Methods

### 2.1. Study Design and Ethics

This cross-sectional study was conducted using an anonymous online questionnaire designed to assess knowledge, clinical practice, diagnostic confidence, and attitudes regarding pediatric oral health and oral mucosal conditions among dentists (DMDs), pediatricians (PEDs), and family/general practitioners (GPs) working in primary healthcare in the Republic of Croatia. The study was conducted in accordance with applicable legal regulations and the principles of the Declaration of Helsinki.

The study protocol was approved by the Ethics Committee of the University of Split School of Medicine (Class: 029-01/25-02/0001; Reg. No.: 2181-198-03-04-25-0010) on 10 February 2025. The study was reported in line with the Checklist for Reporting Results of Internet E-Surveys (CHERRIES) and the STROBE guidelines for cross-sectional studies [36,37].

### 2.2. Participants and Sampling

The target population consisted of dentists, pediatricians, and family/general practitioners actively employed in primary healthcare settings in the Republic of Croatia. Eligible participants were healthcare professionals with a minimum of one year of clinical experience and active involvement in patient care. Retired healthcare professionals and those working exclusively in secondary or tertiary healthcare settings were excluded.

A non-probabilistic sampling approach combining convenience and snowball sampling was used in this study. Invitations were distributed via email and social media platforms, including WhatsApp, Instagram, and Facebook, through the authors’ professional networks and available contact lists. Approximately 1000 emails were sent to dentists, a similar number to general practitioners, and about 150 emails to pediatricians. Participants were also encouraged to forward the survey link to colleagues actively working with pediatric patients. Due to the use of multiple distribution channels and the forwarding of invitations, an exact sample size could not be determined. Also, the use of convenience and snowball sampling may have introduced selection bias, as participants with a greater interest in the topic may have been more likely to respond. The survey was not advertised publicly, and no incentives were offered for participation.

According to national data from 2024, there were 1390 family/general practitioners, 239 pediatricians, and 3434 dentists employed in Croatia [38]. The required sample size was calculated using the Raosoft Sample Size Calculator (RaoSoft^®^, Seattle, WA, USA), assuming a 95% confidence level and a 5% margin of error, resulting in a minimum sample size of 377 participants [39].

### 2.3. Survey Instrument

The questionnaire was specifically designed for this study based on a comprehensive review of the literature related to oral mucosal diseases, diagnostic confidence in oral lesion recognition, and pediatric oral health [10,11,12,13,19,23,26,40,41,42,43,44]. The initial version of the questionnaire was designed by a dentist in collaboration with a specialist in endodontics and restorative dentistry, a specialist in oral medicine, and a specialist in pediatric dentistry. Content validity and clinical relevance were subsequently reviewed and validated by a specialist in oral medicine, contributing to both face and content validity of the questionnaire. Prior to the main survey distribution, the questionnaire was pilot tested on 15 healthcare professionals (7 dentists, 4 pediatricians, and 4 family/general practitioners) to assess clarity, comprehensibility, and completion time. Minor linguistic adjustments were made based on feedback, and no substantial changes to the questionnaire content were required. Internal consistency of the diagnostic confidence scale was assessed using Cronbach’s alpha, which showed good reliability (Cronbach’s α = 0.81). Data from the pilot study participants were not included in the final analysis.

The final questionnaire consisted of 34 questions organized into five thematic sections. The survey was developed and administered in the Croatian language. Most questions were closed-ended, except for age. Likert-type scales were used to assess frequency and diagnostic confidence. The first section (Q1–Q8) collected sociodemographic and professional characteristics of the participants, including profession (dentist, pediatrician, or family/general practitioner), sex, age category, academic background, type of healthcare setting, years of clinical experience, daily patient care hours, and the average number of daily pediatric patient visits. The second section (Q9–Q17) focused on clinical practice related to pediatric oral examination, perceived barriers, and referral patterns for children with oral mucosal lesions. This section included questions addressing routine pediatric oral examination, oral mucosa examination in children, examination and treatment of oral mucosal lesions, reasons for not performing examinations or treatment, and referral practices. Responses in this section were recorded as categorical variables. The third section (Q18–Q20) assessed the self-reported frequency with which participants encountered oral manifestations of systemic diseases, oral mucosal lesions, and oral pain in their clinical practice. Frequency was evaluated using a three-point Likert scale (often, sometimes, rarely). The fourth section (Q21–Q31) evaluated participants’ self-reported diagnostic confidence for common pediatric oral mucosal conditions. Diagnostic confidence was assessed using a five-point Likert scale, where 1 indicated no difficulty in making the diagnosis, 2 minor difficulty, 3 moderate difficulty, 4 major difficulty, and 5 indicated inability to diagnose the condition. This section included infectious, inflammatory, developmental, and traumatic oral mucosal conditions commonly encountered in pediatric patients. The final section (Q32–Q34) examined knowledge, education, and attitudes regarding pediatric oral health. Participants were asked to self-assess their level of knowledge, report previous education related to pediatric oral health, indicate willingness to attend future educational programs, and express their attitudes toward the role of pediatricians and family/general practitioners in the prevention of oral and dental diseases in children. Responses in this section were recorded using categorical scales.

The questionnaire was administered using Google Forms. All questions were mandatory, and only one question was displayed per page. Participants were required to answer all questions before submission and were able to review and modify their responses prior to final submission. No registration or password was required to access the survey, and no technical measures were implemented to prevent duplicate entries.

### 2.4. Data Collection

Data was collected over two months, from March to April 2025. Before completing the questionnaire, participants were informed about the purpose of the study, the number of questions, the estimated completion time, data handling procedures, and the anonymous nature of the survey. Participants were also informed about the inclusion and exclusion criteria. No email addresses or other personally identifiable data were collected. Participation was voluntary and anonymous. Electronically informed consent was obtained before questionnaire completion by selecting a consent option at the beginning of the survey. Participants were free to withdraw from or discontinue the survey at any time before submission. No incentives or rewards were offered for participation.

### 2.5. Data Analysis

Data collected through the online questionnaire were automatically exported to Microsoft Excel (Office 365, 2024, Microsoft, Redmond, WA, USA) and subsequently coded for statistical analysis. Descriptive statistics were used to summarize the data and were presented as absolute numbers and percentages. Differences between dentists, pediatricians, and family/general practitioners were analyzed using the chi-square test or Fisher’s exact test for categorical variables. Ordinal data obtained from Likert-scale questions assessing diagnostic confidence were presented as medians and interquartile ranges and analyzed using the Kruskal–Wallis test. When statistically significant differences were observed, post hoc pairwise comparisons were performed using Dunn’s test with Bonferroni correction. Statistical analyses were performed using IBM SPSS Statistics for Windows, version 29 (IBM Corp., Armonk, NY, USA). A two-sided *p*-value of less than 0.05 was considered statistically significant.

## 3. Results

A total of 632 primary healthcare professionals participated in the study, including 262 dentists (41.5%), 278 family/general practitioners (44.0%), and 92 pediatricians (14.6%). The sample was predominantly female (82.4%). Significant differences were observed between professional groups regarding age distribution, workplace setting, clinical experience, daily working hours, and pediatric patient volume (all *p* < 0.001), as shown in Table 1.

Routine pediatric oral examinations were reported by 96.9% of dentists. Independent treatment of oral mucosal lesions was reported by 75.2% of dentists and 52.2% of pediatricians. Referrals to general dentists were reported by 65.1% of general practitioners, and referrals to oral pathologists were reported by 59.5% of dentists. Other referral pathways were reported by 32.6% of pediatricians (Table 2).

As shown in Table 3, dentists reported rarely encountering oral mucosal lesions and systemic oral manifestations. Pediatricians reported encountering these conditions sometimes. Oral pain was reported more frequently by dentists than by physicians. All reported differences were statistically significant (*p* < 0.001).

As shown in Table 4, reported levels of diagnostic confidence differed between professional groups across most pediatric oral mucosal conditions, with statistically significant differences observed for most conditions (*p* < 0.05). No statistically significant differences between groups were observed for recurrent aphthous ulcerations, geographic tongue, or traumatic oral mucosal injury.

As shown in Table 5, statistically significant differences were observed between professional groups in self-reported knowledge of pediatric oral health (*p* < 0.001) and previous attendance at pediatric oral health courses. Willingness to attend future courses and acknowledgment of the preventive role of pediatricians and family physicians were reported across all groups.

## 4. Discussion

This study examined self-assessed knowledge and diagnostic confidence regarding pediatric oral mucosal lesions among primary healthcare professionals. Dentists reported higher levels of knowledge and diagnostic confidence than pediatricians and family/general practitioners, while physicians more frequently reported limited knowledge and lower confidence. Similar findings have been reported in previous studies, indicating insufficient oral health training among pediatricians and family/general practitioners [23,40,45,46,47]. These differences may be related to variations in educational background and clinical exposure, as dental curricula place strong emphasis on oral mucosal pathology and systematic oral examination [48], whereas medical curricula include limited or no formal training in oral mucosal conditions. Consequently, differences in self-reported knowledge and confidence are more likely attributable to training structure and clinical experience rather than lack of awareness or interest [23,25,34,40,45,46,47,48,49,50,51].

These differences in educational background and clinical exposure were also reflected in self-reported diagnostic confidence. Dentists reported higher confidence in diagnosing most infectious and inflammatory pediatric oral mucosal conditions, including hand, foot, and mouth disease, herpangina, herpetic gingivostomatitis, and candidiasis, whereas general practitioners and pediatricians reported lower confidence levels. Similar patterns have been described in previous studies, which noted limited diagnostic confidence among non-dental healthcare professionals in the assessment of oral mucosal pathology, often associated with reduced training and clinical exposure [10,18,19,20,34]. In contrast, pediatricians and general practitioners reported greater confidence in diagnosing conditions more commonly encountered in neonatal and systemic care, such as congenital epulis and gingival or palatal cysts, likely reflecting their routine clinical exposure [21,24,40,46].

Differences in diagnostic confidence were further mirrored in clinical management and referral practices. Most dentists reported managing pediatric oral mucosal changes independently, while general practitioners and pediatricians reported treating a smaller proportion of cases. When referral was required, pediatricians and general practitioners most frequently referred children to general dentists, whereas dentists more often referred patients to oral pathologists or pediatric dental specialists. This pattern aligns with previous reports indicating that non-dental medical professionals predominantly refer pediatric patients with oral mucosal lesions to general dentists, while dentists demonstrate greater use of specialist referral pathways [19,27,30]. Similar findings have been observed internationally, suggesting that indirect referral pathways remain common and may contribute to delays in definitive diagnosis for conditions with systemic or potentially malignant relevance [26,40,52,53].

In this study, more than two-thirds of pediatricians and family/general practitioners reported routinely examining the oral cavity in children, with higher rates of examination when visible lesions were present. Only a minority reported lack of knowledge or limited time as barriers, which differs from earlier studies identifying insufficient training and time constraints as major obstacles [27,52]. Previous research has shown that despite high reported screening rates, continuing education in pediatric oral health among medical professionals remains limited, which may contribute to gaps in confidence and knowledge [40]. Evidence from scoping reviews further suggests that pediatric oral health knowledge remains insufficient in several key areas, potentially influenced by differences in healthcare systems, educational exposure, and evolving awareness of oral health in recent years [20].

Profession-related differences were also observed in the reported frequency of oral findings encountered in clinical practice. Dentists reported oral manifestations of systemic diseases less frequently than general practitioners and pediatricians, which could be influenced by differences in clinical focus, patient populations, or diagnostic approaches, rather than true differences in occurrence [9,26,51]. Similarly, dentists reported oral mucosal lesions less frequently than medical professionals, despite epidemiological data indicating that such lesions are common in pediatric populations [2,3,4,41,42]. This difference may be related to variation in clinical exposure or recognition of asymptomatic or transient lesions, as previously reported [20,34,46]. Oral pain was reported more frequently by dentists than by medical professionals, although previous studies indicate that oral pain is common in children [20,21,22,34,46]. Collectively, these findings highlight the importance of improving interdisciplinary awareness and strengthening education related to pediatric oral health across both dental and medical care settings.

Self-reported knowledge of children’s oral health was highest among dentists; however, only a minority rated their knowledge as good, while most general practitioners and pediatricians perceived their knowledge as limited. Similar knowledge gaps among medical professionals have been reported previously [20,21,22,25,34,45,51]. Attendance at children’s oral health courses was higher among dentists than among general practitioners and pediatricians, which is consistent with earlier reports describing limited oral health content within medical education curricula [29,30,31,33]. Willingness to attend future educational courses was high across all professional groups, aligning with studies showing strong motivation among healthcare providers to improve oral health competencies when appropriate training opportunities are available [30,31,32,33,52,53].

The observed profession-related differences in reported oral findings, diagnostic confidence, and management practices may have clinical implications for pediatric care. As primary care clinicians and pediatricians frequently encounter oral manifestations of systemic and immune-mediated diseases, improved recognition of early oral signs could support timely referral and management. Previous studies indicate that structured oral health training for primary care clinicians is associated with improvements in knowledge, diagnostic confidence, and clinical practice, supporting the inclusion of oral health education within medical training programs [54,55]. Differences in diagnostic confidence suggest potential benefits of targeted undergraduate and continuing education in pediatric oral pathology, as well as interprofessional educational approaches, which have been shown to enhance confidence and clinical decision-making among pediatric healthcare providers [55]. Furthermore, the observed variation in referral patterns highlights the importance of clear referral pathways and strengthened interdisciplinary collaboration, which may help reduce diagnostic delays and support more consistent integration of oral examinations into pediatric healthcare [54,56]. Collectively, these findings support the further development of interprofessional oral health education and collaborative care models [57,58].

Several limitations should be considered when interpreting the findings of this study. First, the cross-sectional design and use of a self-administered online questionnaire preclude causal inferences. In addition, reliance on self-reported data may have introduced reporting and social desirability bias, as responses reflect perceived rather than objectively assessed knowledge, diagnostic confidence, and clinical practices. The absence of objective assessment methods, such as case-based testing, image interpretation, or direct observation, limits conclusions regarding actual clinical competence. Second, the recruitment strategy based on voluntary participation and non-probability sampling may have affected sample representativeness. Professionals with greater interest in oral health may have been more likely to participate, introducing potential selection bias. Although respondents were recruited from various primary healthcare settings across Croatia, the findings may not be fully generalizable to all primary care professionals. Third, the questionnaire did not differentiate between specific types, severity, or duration of oral mucosal conditions, which may have influenced how participants interpreted questions and reported their clinical experiences. In addition, differences in practice settings, workload, and access to specialist services were not examined and may have affected management and referral patterns. Finally, the study focused on dentists, pediatricians, and family/general practitioners working in primary healthcare, while dental specialists and medical specialists were not included. This limits insight into interdisciplinary differences across levels of care and should be considered when interpreting referral practices.

Despite these limitations, the study provides preliminary insight into self-reported knowledge, diagnostic confidence, and clinical practices related to pediatric oral mucosal conditions among primary healthcare professionals. The findings support the need for further research using objective assessment methods, more representative sampling, and broader professional inclusion to better inform educational strategies and interdisciplinary collaboration in pediatric oral healthcare.

## 5. Conclusions

Considering the limitations of this study, the findings suggest that differences may exist in self-reported preparedness, diagnostic confidence, and clinical management of pediatric oral mucosal lesions among primary healthcare professionals. Dentists generally reported higher confidence and more frequent independent management, whereas pediatricians and family/general practitioners more often relied on referral pathways. These observations should be interpreted with caution, as they are based on self-reported data and may not fully reflect actual clinical competence. Across all professional groups, awareness of the importance of pediatric oral health was high, and most participants expressed willingness to engage in further education. This suggests that observed gaps are likely related to differences in training and clinical exposure rather than lack of interest. The variability in referral practices highlights the potential value of improved interdisciplinary collaboration, clearer clinical guidance, and targeted educational initiatives to support primary healthcare professionals in the early recognition and management of pediatric oral mucosal conditions.

## Figures and Tables

**Table 1:** Sociodemographic and professional characteristics of study participants.

Variable	Characteristics	DMD N (%)	GP-MD N (%)	PED-MD N (%)	*p*-Value
Sex	Female	223 (85.1)	228 (82.0)	70 (76.1)	0.001 *
	Male	39 (14.9)	50 (18.0)	22 (23.9)	
Age category (years)	<35	120 (45.8)	53 (19.1)	2 (2.2)	0.001 *
	35–44	57 (21.8)	125 (45.0)	9 (9.8)	
	44–54	49 (18.7)	57 (20.4)	32 (34.8)	
	>55	36 (13.7)	40 (15.5)	49 (53.2)	
Academic background	DMD/DM	243 (92.7)	239 (86.0)	60 (65.2)	0.001 *
	MSc	7 (2.7)	16 (5.8)	21 (22.8)	
	PhD	12 (4.6)	23 (8.2)	11 (12.0)	
Types of health care settings	Health center	110 (42.0)	235 (84.5)	48 (52.2)	0.001 *
	Private practice under concession	66 (25.2)	37 (13.3)	38 (41.3)	
	Private practice	86 (32.8)	6 (2.2)	6 (6.5)	
Years of clinical experience	1–5	99 (37.8)	119 (42.8)	0 (0)	0.001 *
	6–10	41 (15.6)	41 (14.7)	11 (12.1)	
	11–20	49 (18.7)	66 (23.7)	47 (51.0)	
	>20	73 (27.9)	52 (18.7)	34 (36.9)	
Daily patient care hours	≤8	234 (89.3)	215 (77.3)	60 (65.2)	0.001 *
	>8	28 (10.7)	63 (22.7)	32 (34.8)	
Daily pediatric patient visit	<10	242 (92.4)	147 (52.9)	3 (3.3)	0.001 *
	11–20	18 (7.6)	74 (26.6)	44 (47.8)	
	21–50	0 (0)	36 (12.9)	18 (19.6)	
	>50	0 (0)	21 (7.6)	27 (29.3)	

Abbreviations: DMD, Dentists; GP-MD, Family/General Practitioners; PED-MD, Pediatricians. Data are presented as frequencies (percentages). Chi-square test or Fisher’s exact test, * *p* < 0.05.

**Table 2:** Clinical practice, barriers, and referral patterns related to pediatric oral mucosa examination.

Variable	Characteristics	DMD N (%)	GP-MD N (%)	PED-MD N (%)	*p*-Value
Routine pediatric oral examination	Yes	254 (96.9)	214 (77.0)	74 (80.4)	0.001 *
Examination of children with oral mucosal lesions (diseases)	Yes	252 (96.2)	234 (84.2)	70 (76.1)	0.001 *
Treatment of oral mucosal lesions in children	Yes	197 (75.2)	197 (70.9)	48 (52.2)	0.001 *
Reason for not performing oral mucosa examination	Lack of knowledge	8 (3.1)	21 (7.6)	15 (16.3)	0.001 *
	Lack of time	8 (3.1)	48 (17.3)	16 (17.4)	
	Lack of interest	0 (0)	0 (0)	0 (0)	
	Other	246 (93.8)	209 (75.2)	61 (66.3)	
Reason for not treating oral mucosal lesions	Lack of knowledge	40 (15.3)	57 (20.5)	27 (29.3)	0.001 *
	Lack of time	5 (1.9)	26 (9.4)	12 (13.0)	
	Lack of interest	0 (0)	0 (0)	2 (2.2)	
	Other	217 (82.8)	195 (70.0)	51 (55.4)	
Referral of children with oral mucosal lesions (multiple answers possible)	General dentist	0 (0)	181 (65.1)	50 (54.3)	0.001 *
	Pediatric dentist	101 (38.5)	3 (1.1)	7 (7.6)	0.001 *
	Oral pathologist	156 (59.5)	43 (15.5)	2 (2.2)	0.001 *
	Oral surgeon	17 (6.5)	27 (9.7)	0 (0)	0.006 *
	Dermatologist	5 (1.9)	0 (0)	1 (1.1)	0.001 *
	Infectious disease specialist	4 (1.5)	1 (0.4)	2 (2.2)	0.267
	Pediatrician	41 (15.6)	14 (5.3)	0 (0)	0.001 *
	Family physician	4 (1.5)	0 (0)	0 (0)	0.001 *
	Other	0 (0)	0 (0)	30 (32.6)	0.001 *

Abbreviations: DMD, Dentists; GP-MD, Family/General Practitioners; PED-MD, Pediatricians. Data are presented as frequencies (percentages). Chi-square test or Fisher’s exact test, * *p* < 0.05.

**Table 3:** Self-reported frequency of oral findings encountered in clinical practice.

Variable	Characteristics	DMD N (%)	GP-MD N (%)	PED-MD N (%)	*p*-Value
Oral manifestations of systemic diseases	Rarely	206 (78.6)	150 (54.0)	40 (43.5)	0.001 *
	Sometimes	46 (17.6)	107 (38.5)	49 (53.3)	
	Often	10 (3.8)	21 (7.6)	3 (3.2)	
Oral mucosal lesions	Rarely	184 (70.2)	120 (43.2)	33 (35.9)	0.001 *
	Sometimes	73 (27.9)	125 (45.0)	52 (55.4)	
	Often	5 (1.9)	33 (11.9)	8 (8.7)	
Oral pain	Rarely	87 (33.2)	132 (47.5)	46 (50.0)	0.001 *
	Sometimes	99 (37.8)	107 (38.5)	34 (37.0)	
	Often	76 (29.0)	39 (14.0)	12 (13.0)	

Abbreviations: DMD, Dentists; GP-MD, Family/General Practitioners; PED-MD, Pediatricians. Data are presented as frequencies (percentages). Chi-square test or Fisher’s exact test, * *p* < 0.05.

**Table 4:** Self-reported diagnostic confidence for pediatric oral mucosal conditions.

Oral Mucosal Condition	DMD	GP-MD	PED-MD	*p*-Value
Gingival and palatal cyst	2.37 ± 1.21 ^a,b^	2.74 ± 1.21 ^a^	2.76 ±1.16 ^b^	0.001 *
Congenital epulis	2.83 ± 1.36 ^a,b^	3.59 ± 1.52 ^a,c^	3.25 ± 1.30 ^b,c^	0.001 *
Streptococcal stomatitis	2.73 ± 1.22 ^a,b^	2.40 ± 1.22 ^a^	2.40 ±1.11 ^b^	0.003 *
Hand, foot, and mouth disease	2.67 ± 1.28 ^a,b^	1.85 ± 1.23 ^a^	2.03 ±1.22 ^b^	0.001 *
Herpetic gingivostomatitis	2.13 ± 1.37	1.91 ± 1.15 ^a^	2.16 ±1.16 ^a^	0.043 *
Herpangina	2.60 ± 1.17 ^a,b^	1.83 ± 1.09 ^a^	1.98 ±1.12 ^b^	0.001 *
Acute atrophic candidiasis	2.51 ± 1.24 ^a^	2.48 ± 1.05 ^b^	2.21 ± 1.19 ^a,b^	0.048 *
Acute pseudomembranous candidiasis	2.31 ± 1.34 ^a,b^	1.87 ± 1.21 ^a^	1.98 ± 1.21 ^b^	0.001 *
Recurrent aphthous ulcerations	2.14 ± 1.47	2.00 ± 1.26	2.22 ± 1.24	0.186
Geographic tongue	2.02 ± 1.55	1.94 ± 1.27	2.16 ± 1.33	0.059
Traumatic oral mucosal injury	2.01 ± 1.48	1.85 ± 1.23	1.85 ± 1.22	0.960

Abbreviations: DMD, Dentists; GP-MD, Family/General Practitioners; PED-MD, Pediatricians. Data are presented as mean (standard deviation). Differences between groups were analyzed using the Kruskal–Wallis test. Post hoc pairwise comparisons were performed using Dunn’s test with Bonferroni correction. Within a row, the same letter indicates a statistically significant difference between groups. * *p* < 0.05.

**Table 5:** Knowledge, education, and attitudes regarding pediatric oral health.

Variable	Characteristics	DMD N (%)	GP-MD N (%)	PED-MD N (%)	*p*-Value
Self-assessed knowledge of pediatric oral health	Poor	89 (34.0)	191 (68.7)	56 (60.9)	0.001 *
	Moderate	104 (39.7)	65 (23.4)	30 (32.6)	
	Good	49 (26.3)	22 (7.9)	6 (6.5)	
Attendance at pediatric oral health courses	Yes	163 (62.2)	44 (15.8)	14 (15.2)	0.001 *
Willingness to attend future pediatric oral health courses	Yes	210 (80.2)	234 (84.2)	69 (75.0)	0.128
Perceived role of pediatricians and family physicians in prevention of pediatric oral diseases	Yes	251 (96.2)	248 (89.2)	72 (78.3)	0.001 *

Abbreviations: DMD, Dentists; GP-MD, Family/General Practitioners; PED-MD, Pediatricians. Data are presented as frequencies (percentages). Chi-square test or Fisher’s exact test, * *p* < 0.05.

## Data Availability

The original contributions presented in this study are included in the article. Further inquiries can be directed to the corresponding author.
